# User-Centered Development of a Patient Decision Aid for Choice of Early Abortion Method: Multi-Cycle Mixed Methods Study

**DOI:** 10.2196/48793

**Published:** 2024-04-16

**Authors:** Kate J Wahl, Melissa Brooks, Logan Trenaman, Kirsten Desjardins-Lorimer, Carolyn M Bell, Nazgul Chokmorova, Romy Segall, Janelle Syring, Aleyah Williams, Linda C Li, Wendy V Norman, Sarah Munro

**Affiliations:** 1 Department of Obstetrics and Gynecology University of British Columbia Vancouver, BC Canada; 2 Department of Obstetrics and Gynecology Dalhousie University Halifax, NS Canada; 3 Department of Health Systems and Population Health School of Public Health University of Washington Seattle, WA United States; 4 Department of Family Practice University of British Columbia Vancouver, BC Canada; 5 Department of Physical Therapy University of British Columbia Vancouver, BC Canada; 6 Department of Public Health, Environments and Society Faculty of Public Health and Policy London School of Hygiene & Tropical Medicine London United Kingdom

**Keywords:** family planning, abortion, shared decision-making, patient decision aid, qualitative, evaluation, Canada, health equity

## Abstract

**Background:**

People seeking abortion in early pregnancy have the choice between medication and procedural options for care. The choice is preference-sensitive—there is no clinically superior option and the choice depends on what matters most to the individual patient. Patient decision aids (PtDAs) are shared decision-making tools that support people in making informed, values-aligned health care choices.

**Objective:**

We aimed to develop and evaluate the usability of a web-based PtDA for the Canadian context, where abortion care is publicly funded and available without legal restriction.

**Methods:**

We used a systematic, user-centered design approach guided by principles of integrated knowledge translation. We first developed a prototype using available evidence for abortion seekers’ decisional needs and the risks, benefits, and consequences of each option. We then refined the prototype through think-aloud interviews with participants at risk of unintended pregnancy (“patient” participants). Interviews were audio-recorded and documented through field notes. Finally, we conducted a web-based survey of patients and health care professionals involved with abortion care, which included the System Usability Scale. We used content analysis to identify usability issues described in the field notes and open-ended survey questions, and descriptive statistics to summarize participant characteristics and close-ended survey responses.

**Results:**

A total of 61 individuals participated in this study. Further, 11 patients participated in think-aloud interviews. Overall, the response to the PtDA was positive; however, the content analysis identified issues related to the design, language, and information about the process and experience of obtaining abortion care. In response, we adapted the PtDA into an interactive website and revised it to include consistent and plain language, additional information (eg, pain experience narratives), and links to additional resources on how to find an abortion health care professional. In total, 25 patients and 25 health care professionals completed the survey. The mean System Usability Scale score met the threshold for good usability among both patient and health care professional participants. Most participants felt that the PtDA was user-friendly (patients: n=25, 100%; health care professionals: n=22, 88%), was not missing information (patients: n=21, 84%; health care professionals: n=18, 72%), and that it was appropriate for patients to complete the PtDA before a consultation (patients: n=23, 92%; health care professionals: n=23, 92%). Open-ended responses focused on improving usability by reducing the length of the PtDA and making the website more mobile-friendly.

**Conclusions:**

We systematically designed the PtDA to address an unmet need to support informed, values-aligned decision-making about the method of abortion. The design process responded to a need identified by potential users and addressed unique sensitivities related to reproductive health decision-making.

## Introduction

In total, 1 in 3 pregnancy-capable people in Canada will have an abortion in their lifetimes, and most will seek care early in pregnancy [1]. Medication abortion (using the gold-standard mifepristone/misoprostol regimen) and procedural abortion are common, safe, and effective options for abortion care in the first trimester [2,3]. The choice between using medications and presenting to a facility for a procedure is a preference-sensitive decision; there is no clinically superior option and the choice depends on what matters most to the individual patient regarding the respective treatments and the features of those options [4-6].

The choice of method of abortion can involve a process of shared decision-making, in which the patient and health care professional share the best available evidence about options, and the patient is supported to consider those options and clarify an informed preference [7]. There are many types of interventions available to support shared decision-making, including interventions targeting health care professionals (eg, educational materials, meetings, outreach visits, audit and feedback, and reminders) and patients (eg, patient decision aids [PtDA], appointment preparation packages, empowerment sessions, printed materials, and shared decision-making education) [8]. Of these interventions, PtDAs are well-suited to address challenges to shared decision-making about the method of abortion, including limited patient knowledge, public misinformation about options, poor access to health care professionals with sufficient expertise, and apprehension about abortion counseling [9].

PtDAs are widely used interventions that support people in making informed, deliberate health care choices by explicitly describing the health problem and decision, providing information about each option, and clarifying patient values [10]. The results of the 2023 Cochrane systematic review of 209 randomized controlled trials indicate that, compared to usual care (eg, information pamphlets or webpages), the use of PtDAs results in increases in patient knowledge, expectations of benefits and harms, clarity about what matters most to them, and participation in making a decision [11]. Of the studies included in the systematic review, 1 tested the effect of a PtDA leaflet for method of abortion and found that patients eligible for both medication and procedural abortion who received the PtDA were more knowledgeable, and had lower risk perceptions and decisional conflict than those who were in the control group [12]. However, that PtDA was developed 20 years ago in the UK health system and was not publicly available. A recent environmental scan of PtDAs for a method of abortion found that other available options meet few of the criteria set by the International Patient Decision Aid Standards (IPDAS) collaboration and do not include language and content optimized for end users [9,13].

Consequently, no PtDAs for method of abortion were available in Canada at the time of this study. This was a critical gap for both patients and health care professionals as, in 2017, mifepristone/misoprostol medication abortion came to the market, offering a new method of choice for people seeking abortion in the first trimester [14]. Unlike most jurisdictions, in Canada medication abortion is typically prescribed in primary care and dispensed in community pharmacies. Offering a PtDA in preparation for a brief primary care consultation allows the person seeking abortion more time to digest new information, consider their preferences, be ready to discuss their options, and make a quality decision.

In this context, we identified a need for a high-quality and publicly available PtDA to support people in making an informed choice about the method of abortion that reflects what is most important to them. Concurrently, our team was working in collaboration with knowledge users (health care professionals, patients, and health system decision makers) who were part of a larger project to investigate the implementation of mifepristone in Canada [15,16]. We, therefore, aimed to develop and evaluate the usability of a web-based PtDA for the Canadian context, where abortion care is publicly funded and available without legal restriction.

## Methods

### Study Design

We performed a mixed methods user-centered development and evaluation study informed by principles of integrated knowledge translation. Integrated knowledge translation is an approach to collaborative research in which researchers and knowledge users work together to identify a problem, conduct research as equal partners to address that problem, and coproduce research products that aim to impact health service delivery [17]. We selected this approach to increase the likelihood that our end PtDAs would be relevant, useable, and used for patients and health care professionals in Canada [17]. The need for a PtDA was identified through engagement with health care professionals. In 2017, they highlighted the need for patients to be supported in choosing between procedural care—which historically represented more than 90% of abortions in Canada [18]—and the newly available medication option [19,20]. This need was reaffirmed in 2022 by the Canadian federal health agency, Health Canada, which circulated a request for proposals to generate “evidence-based, culturally-relevant information aimed at supporting people in their reproductive decision-making and in accessing abortion services as needed” [21].

We operationalized integrated knowledge translation principles in a user-centered design process. User-centered design “grounds the characteristics of an innovation in information about the individuals who use that innovation, with a goal of maximizing ‘usability in context’” [22]. In PtDA development, user-centered design involves iteratively understanding users, developing and refining a prototype, and observing user interaction with the prototype [23,24]. Like integrated knowledge translation, this approach is predicated on the assumption that involving users throughout the process increases the relevance of the PtDA and the likelihood of successful implementation [24].

**Figure 1:**
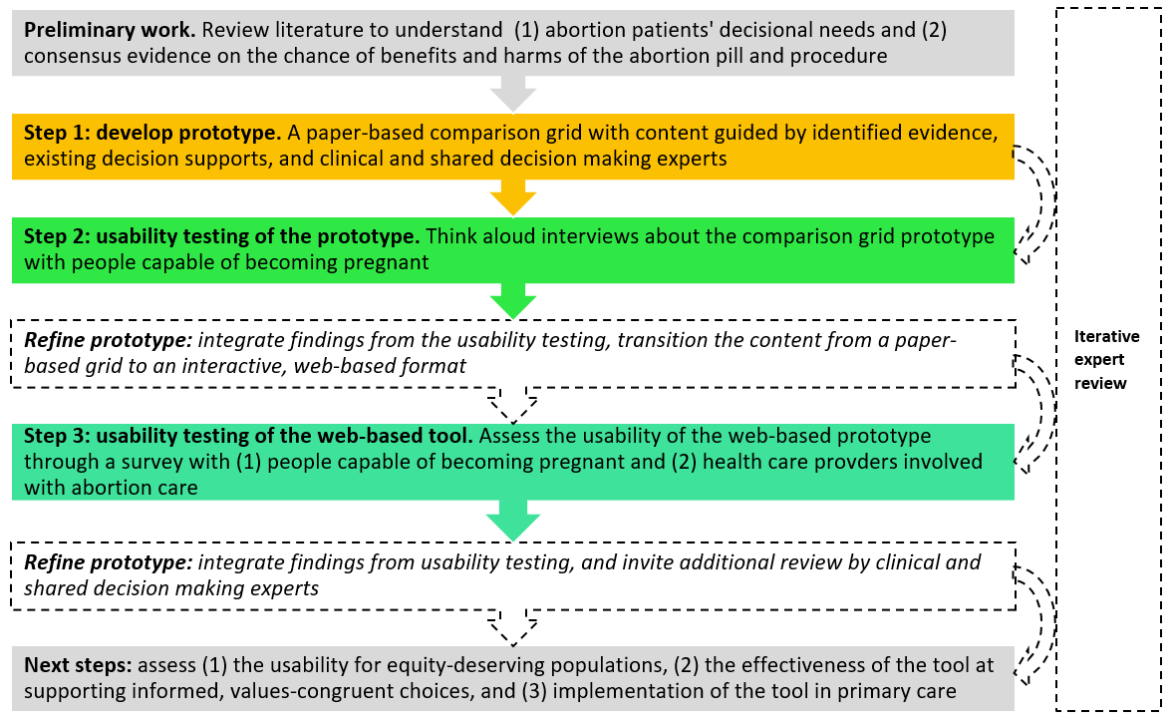
PtDA development process. PtDA: patient decision aid.

Our design process included the following steps (Figure 1): identification of evidence about abortion patients’ decisional needs and the attributes of medication and procedural abortion that matter most from a patient perspective; development of a paper-based prototype; usability testing via think-aloud interviews with potential end users; refinement of the PtDA prototype into an interactive website; usability testing via a survey with potential end users and abortion health care professionals; and final revisions before launching the PtDA for real-world testing. Our systematic process was informed by user-centered methods for PtDA development [23,24], guidance from the IPDAS collaboration [25-27], and the Standards for Universal Reporting of Patient Decision Aid Evaluation checklist [10].

Our multidisciplinary team included experts in shared decision-making (SM and LT), a PhD student in patient-oriented knowledge translation (KJW), experts in integrated knowledge translation with health care professionals and policy makers (WVN and SM), clinical experts in abortion counseling and care (WVN and MB), a medical undergraduate student (RS), a research project coordinator (AW), and family medicine residents (KD-L, CMB, NC, and JS) who had an interest in abortion care. Additionally, a panel of experts external to the development process reviewed the PtDA for clinical accuracy following each revision of the prototype. These experts included coauthors of the national Society for Obstetricians and Gynaecologists of Canada (SOGC) clinical practice guidelines for abortion care in Canada. They were invited to this project because of their knowledge of first-trimester abortion care as well as their ability to support the implementation of the PtDA in guidelines and routine clinical practice.

### Ethical Considerations

The research was approved by the University of British Columbia Children’s and Women’s Research Ethics Board (H16-01006) and the Nova Scotia Health Research Ethics Board (1027637). In each round of testing, participants received a CAD $20 (US $14.75) Amazon gift card by email for their participation.

### Preliminary Work: Identification of Evidence

We identified the decisional needs of people seeking early abortion care using a 2018 systematic review of reasons for choosing an abortion method [28], an additional search that identified 1 study conducted in Canada following the 2017 availability of mifepristone/misoprostol medication abortion [29], and the SOGC clinical practice guidelines [2,3]. The review identified several key factors that matter most for patient choice of early abortion method: *perceived simplicity and “naturalness,”* *fear of complication or bleeding*, *fear of anesthesia or surgery*, *timing of the procedure*, and *chance of sedation*. The additional Canadian study found that the *time required to complete the abortion* and *side effects* were important factors. According to the SOGC clinical practice guidelines, the key information that should be communicated to the patient are *gestational age limits* and *the risk of complications with increasing gestational age* [2,3]. The guidelines also indicate that *wait times*, *travel times*, and *cost considerations* may be important in a person’s choice of abortion method and should be addressed [2,3].

We compiled a long list of attributes for our expert panel and then consolidated and refined the attribute list through each stage of the prototype evaluation. For evidence of how these factors differed for medication and procedural abortion, we drew primarily from the SOGC clinical practice guidelines for abortion [2,3]. For cost considerations, we described the range of federal, provincial, and population-specific programs that provide free coverage of abortion care for people in Canada.

### Step 1: Developing the Prototype

Our goal was to produce an interactive, web-based PtDA that would be widely accessible to people seeking an abortion in Canada by leveraging the widespread use of digital health information, especially among reproductive-aged people [30]. Our first prototype was based on a previously identified paper-based question-and-answer comparison grid that presented evidence-based information about the medication and procedural options [9,31]. We calculated readability by inputting the plain text of the paper-based prototype into a Simple Measure of Gobbledygook (SMOG) Index calculator [32].

We made 2 intentional deviations from common practices in PtDA development [33]. First, we did not include an “opt-out” or “do nothing” option, which would describe the natural course of pregnancy. We chose to exclude this option to ensure clarity for users regarding the decision point; specifically, our decision point of interest was the method of abortion, not the choice to terminate or continue a pregnancy. Second, we characterized attributes of the options as key points rather than positive and negative features to avoid imposing value judgments onto subjective features (eg, having the abortion take place at home may be beneficial for some people but may be a deterrent for others).

### Step 2: Usability Testing of the Prototype

We first conducted usability testing involving think-aloud interviews with patient participants to assess the paper-based prototype. Inclusion criteria included people aged 18-49 years assigned-female-at-birth who resided in Canada and could speak and read English. In January 2020, we recruited participants for the first round of think-aloud interviews [34] via email and poster advertising circulated to (1) a network of parent research advisors who were convened to guide a broader program of research about pregnancy and childbirth in British Columbia, Canada, and (2) a clinic providing surgical abortion care in Nova Scotia, Canada, as well as snowball sampling with participants. We purposively sought to advertise this study with these populations to ensure variation in age, ethnicity, level of education, parity, and abortion experience. Interested individuals reviewed this study information form and provided consent to participate, before scheduling an interview. The interviewer asked participants to think aloud as they navigated the prototype, for example describing what they liked or disliked, missing information, or lack of clarity. The interviewer noted the participant’s feedback on a copy of the prototype during the interview. Finally, the participant responded to questions adapted from the System Usability Scale [35], a measure designed to collect subjective ratings of a product’s usability, and completed a brief demographic questionnaire. The interviews were conducted via videoconferencing and were audio recorded. We deidentified the qualitative data and assigned each participant a unique identifier. Then, the interviewer listened to the recording and revised their field notes with additional information including relevant quotes.

For the analysis of think-aloud interviews, we used inductive content analysis to describe the usability and acceptability of different elements of the PtDA [36]. Further, 3 family medicine residents (KD-L, CMB, and NC) under guidance from a senior coauthor (SM) completed open coding to develop a list of initial categories, which we grouped under higher-order headings. We then organized these results in a table to illustrate usability issues (categories), illustrative participant quotes, and modifications to make. We then used the results of interviews to adapt the prototype into a web-based format, which we tested via further think-aloud interviews and a survey with people capable of becoming pregnant and health care professionals involved with abortion care.

### Step 3: Usability Testing of the Website

For the web-based format, we used DecideApp PtDA open-source software, which provides a sustainable solution to the problems of low quality and high maintenance costs faced by web-based PtDAs by allowing developers to host, maintain, and update their tools at no cost. This software has been user-tested and can be accessed by phone, tablet, or computer [37,38]. It organizes a PtDA into 6 sections: Introduction, About Me, My Values, My Choice, Review, and Next Steps. In the My Values section, an interactive values clarification exercise allows users to rank and make trade-offs between attributes of the options. The final pages provide an opportunity for users to make a choice, complete a knowledge self-assessment, and consider the next steps to access their chosen method.

From July to August 2020, we recruited patient and health care professional participants using Twitter and the email list of the Canadian Abortion Providers Support platform, respectively. Participants received an email with a link to the PtDA and were redirected to the survey once they had navigated through the PtDA. As above, inclusion criteria included people aged 18-49 years assigned as female-at-birth who resided in Canada. Among health care professionals, we included eligible prescribers who may not have previously engaged in abortion care (family physicians, residents, nurse practitioners, and midwives), and allied health professionals and stakeholders who provide or support abortion care, who practiced in Canada. All participants had to speak and read English.

The survey included 3 sections: usability, implementation, and participant characteristics. The usability section consisted of the System Usability Scale [35], and purpose-built questions about what participants liked and disliked about the PtDA. The implementation section included open- and close-ended questions about how the PtDA compares to other resources and when it could be implemented in the care pathway. Patient participants also completed the Control Preference Scale, a validated measure used to determine their preferred role in decision-making (active, collaborative, or passive) [39]. Data on participant characteristics included gender, abortion experience (patient participants), and abortion practice (health care professional participants). We deidentified the qualitative data and assigned each participant a unique identifier. For the analysis of survey data, we characterized close-ended responses using descriptive statistics, and, following the analysis procedures described in Step 2 in the Methods section, used inductive content analysis of open-ended responses to generate categories associated with usability and implementation [36]. In 2021, we made minor revisions to the website based on the results of usability testing and published the PtDA for use in routine clinical care.

## Results

### Overview

In the following sections, we outline the results of the development process including the results of the think-aloud interviews and survey, as well as the final decision aid prototype.

### Step 1: Developing the Prototype

Our initial prototype, a paper-based question-and-answer comparison grid, presented evidence-based information comparing medication and procedural abortion. The first version of the prototype also included a second medication abortion regimen involving off-label use of methotrexate, however, we removed this option following a review by the clinical expert panel who advised us that there is very infrequent use of this regimen in Canada in comparison to the gold standard medication abortion option, mifepristone. Other changes at this stage involved clarifying the scope of practice (health care professionals other than gynecologists can perform a procedural abortion), abortion practice (gestational age limit and how the medication is taken), the abortion experience (what to expect in terms of bleeding), and risk (removing information about second- and third-trimester abortion). The updated prototype was finalized by a scientist (SM) and trainee (KJW) with expertise in PtDA development. The prototype (see Multimedia Appendix 1) was ultimately 4 pages long and described 18 attributes of each option framed as Frequently Asked Questions, including abortion eligibility (How far along in pregnancy can I be?), duration (How long does it take?), and side effects (How much will I bleed?). The SMOG grade level was 8.4.

### Step 2: Usability Testing of the Prototype

#### Participant Characteristics

We included 11 participants in think-aloud interviews between January and July 2020, including 7 recruited through a parent research advisory network and 4 individuals who had recently attended an abortion clinic. The mean interview duration was 36 minutes (SD 6 minutes). The participants ranged in age from 31 to 37 years. All had been pregnant and 8 out of 11 (73%) participants had a personal experience of abortion (4 participants who had recently attended an abortion clinic and 4 participants from the parent research advisory who disclosed their experience during the interview). The characteristics of the sample are reported in Table 1.

**Table 1:** Characteristics of think-aloud interview participants (N=11).

Characteristic		Participants, n (%)
**Gender**		
	Cisgender woman	11 (100)
	Pregnant ever	11 (100)
**Race**		
	Black	1 (9)
	Chinese	1 (9)
	Hispanic or Latin American	3 (27)
	South Asian	1 (9)
	White	5 (46)
**Education**		
	College	2 (18)
	University	9 (82)

#### Usability

Overall, participants had a positive view of the paper-based, comparison grid PtDA. In total, 1 participant who had recently sought an abortion said, “I think this is great and super helpful. It would’ve been awesome to have had access to this right away … I don’t think there’s really anything missing from here that I was Googling about” (DA010). The only participant who expressed antichoice views indicated that the PtDA would be helpful to someone seeking to terminate a pregnancy (DA001). Another participant said, “[The PtDA] is not biased, it’s not like you’re going to die. It’s a fact, you know the facts and then you decide whether you want it or not. A lot of people feel it’s so shameful and judgmental, but this is very straightforward. I like it.” (DA002). Several participants stated they felt more informed and knowledgeable about the options.

In response to questions adapted from the System Usability Scale, all 11 participants agreed that the PtDA was easy to use, that most people could learn to use it quickly, and that they felt very confident using the prototype, and disagreed that it was awkward to use. In total, 8 (73%) participants agreed with the statement that the components of the PtDA were well-integrated. A majority of participants disagreed with the statements that the website was unnecessarily complex (n=8, 73%), that they would need the support of an expert to use it (n=8, 73%), that it was too inconsistent (n=9, 82%), and that they would need to learn a lot before using it (n=8, 73%). Further, 2 (18%) participants agreed with the statements that the PtDA was unnecessarily complex and that they would need to learn a lot before using it. Furthermore, 1 (9%) participant agreed with the statement that the PtDA was too inconsistent.

Through inductive analysis of think-aloud interviews, we identified 4 key usability categories: design, language, process, and experience.

#### Design

Participants liked the side-by-side comparison layout, appreciated the summary of key points to remember, and said that overall, the presented information was clear. For example, 1 participant reflected, “I think it’s very clear ... it’s very simplistic, people will understand the left-hand column is for medical abortion and the right-hand column is for surgical.” (DA005) Some participants raised concerns about the aesthetics of the PtDA, difficulties recalling the headers across multiple pages, and the overall length of the PtDA.

#### Language

Participants sought to clarify language at several points in the PtDA. Common feedback was that the gestational age limit for the medication and the procedure should be clarified. Participants also pointed out inconsistent use of language (eg, doctor and health care professional) and medical jargon.

#### Process

Several participants were surprised to learn that family doctors could provide abortion care. Others noted that information about the duration—including travel time—and number of appointments for both medication and procedural abortion could be improved. In addition to clarifying the abortion process, several participants suggested including additional information and resources to help identify an abortion health care professional, understand when to seek help for abortion-related complications, and access emotional support. It was also important to participants that financial impacts (eg, hospital parking and menstrual pads) were included for each option.

#### Experience

Participants provided insight into the description of the physical, psychological, and other consequences associated with the abortion medication and procedure. Participants who had both types of abortion care felt that the description of pain that “may be worse than a period” was inaccurate. Other participants indicated that information about perceived and real risks was distressing or felt out of place, such as correcting myths about future fertility or breast cancer. Some participants indicated that patient stories would be valuable saying, for example, “I think what might be nice to help with the decision-making process is reading stories of people’s experiences” (DA006).

#### Modifications Made

Changes made based on these findings are described in Table 2. Key user-centered modifications included transitioning to a web-based format with a consistent color scheme, clarifying who the PtDA is for (for typical pregnancies up to 10 weeks), adding information about telemedicine to reflect guidelines for the provision of abortion during pandemics, and developing brief first-person qualitative descriptions of the pain intensity for each option.

Through analysis of the interviews and consultation with our panel of clinical experts, we also identified that, among the 18 initial attributes in our prototype, 7 had the most relative importance to patients in choosing between medication and procedural abortion. These attributes also represented important differences between each option which forced participants to consider the trade-offs they were willing to make. Thus we moved all other potential attributes into an information section (My Options) that supported the user to gain knowledge before clarifying what mattered most to them by considering the differences between options (My Values).

**Table 2:** Usability issues identified by participants in think-aloud interviews and resulting changes.

Issue and illustrative participant quotes		Modifications made
**Design**		
	“I assume it will be formatted a bit more attractively?” (DA003)	Transitioned to web-based format with a consistent color scheme
	“My question is about what the format will be…it is spread over multiple pages, people may need the [headers] again to understand.” (DA005)	Divided comparison grid into 3 sections (how it works, how it feels, and key points to remember)
	“The longer you make a document, the less people are going to read it anyway, they are just going to start to skim.” (DA009)	Reviewed for succinctness Broke up the content into separate sections for qualitative pain descriptions
**Language**		
	“When I am looking at that point [about how far along in pregnancy I can be], I’m not sure if just doesn’t click immediately…it is a little bit confusing.” (DA011)	Clarified that the information in the PtDA^a^ is for typical pregnancies up to 10 weeks
	“Review it with the lens of only using different language where there is something different to be said.” (DA003)	Reviewed for consistent terminology
	“It seems like it’s a bunch of medical jargon...I would want it to actually say…pill [rather than medication names].” (DA005)	Changed terminology to “abortion pill” and “abortion procedure”
**Process**		
	“I found that confusing, because when I went to my family doctor, she told me to go to [the hospital] in order to make either decision…but my family doctor could have just prescribed me the pill?” (DA008)	Added information: patients can ask their family doctor if they provide the abortion pill
	“I was there [at the hospital] at 7, I didn’t know it was going to be a 4-hour procedure …it’s a lot longer than 5-10 minutes…it doesn’t really say you will be at the hospital for a few hours…you need half a day off if you’re going the hospital route.” (DA008)	Clarified time off is needed on the day of the procedure Added information about telemedicine (reflection of SOGC^b^ guidelines for the provision of abortion during pandemics)
	“Why breast cancer? I would worry about cervical or uterine cancer, the actual place of the procedure.” (DA004) “I guess you have to put the associated risks…could that be provided once they made the decision?” (DA003)	Removed statement saying there is no increased risk of breast cancer, which reflected a common misconception in the United States that abortion causes malignancy [40] Rephrased some of the risk language (eg, removed the term “perforation”)
	“Is this tool going to provide a list of clinics or the hospitals that do provide it?” (DA006)	Added links to external resources about how to find an abortion health care professional
**Experience**		
	“Just a comment on the pain: it feels like you’re giving birth, it feels like you’re going through labour…I think this doesn’t really give you the full picture. It's painful, it’s extremely painful.” (DA006) “Gentle [suction] my ass.” (DA009) “I’d say worse than period-like cramps, but of course everybody is different.” (DA011)	Developed and added brief first-person qualitative descriptions (mean 47 words, SD 8.3 words) of how low-, medium-, and high-intensity pain could feel for each option (descriptions were synthesized from 58 published accounts of abortion care, and designed them to be similar in length and structure to minimize bias toward either option—see Multimedia Appendix 2)
	“I can imagine how traumatic it would be…what kind of support would be available?” (DA001)	Clarified that abortion is not associated with an increased risk of mental illness Added a list of resources for users to speak with someone about their mental health and abortion care

^a^PtDA: patient decision aid.

^b^SOGC: Society of Obstetricians and Gynaecologists of Canada.

### Step 3: Usability Testing of the Website

#### Description of the PtDA

**Figure 2:**
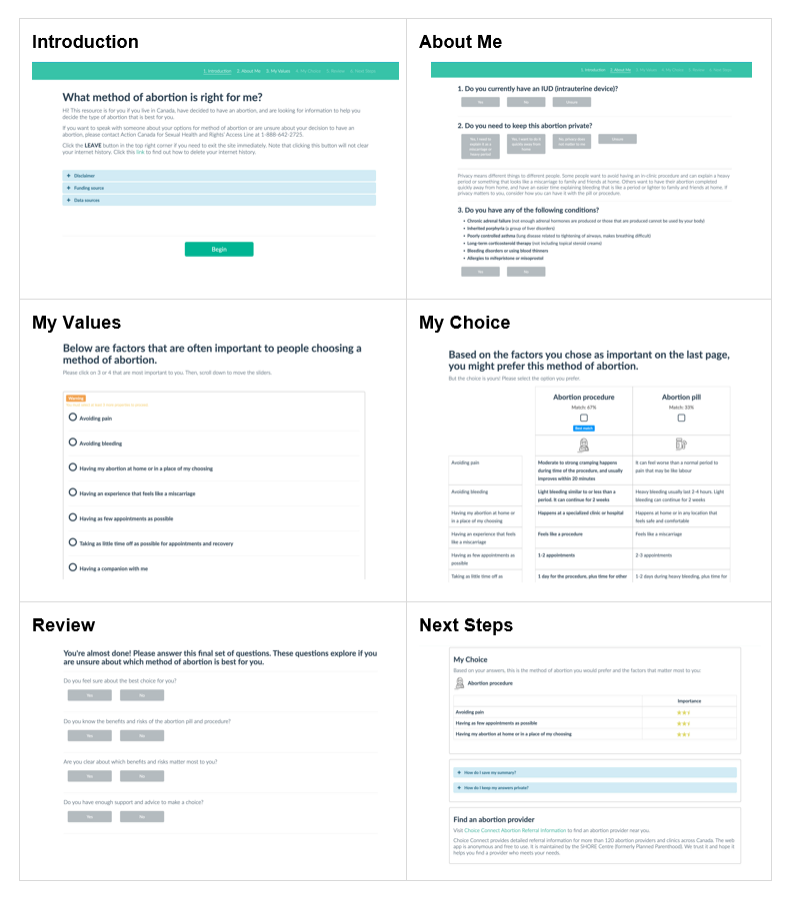
*It’s My Choice* patient decision aid web app.

As shown in Figure 2, the revised version of the PtDA resulting from our systematic process is an interactive website. Initially, the title was *My Body, My Choice*; however, this was changed to avoid association with antivaccine campaigns that co-opted this reproductive rights slogan. The new title, *It’s My Choice* or *C’est Mon Choix*, was selected for its easy use in English and French. The PtDA leads the user through 6 sections:

- The *Introduction* section provides the user with information about the decision and the PtDA, as well as grids comparing positive and negative features of the abortion pill and procedure, including their chance of benefits (eg, effectiveness), harms (eg, complications), and other relevant factors (eg, number of appointments and cost).
- The *About Me* section asks the user to identify any contraindications to the methods. It then prompts users to consider their privacy needs and gives examples of how this relates to each option (eg, the abortion pill can be explained to others as a miscarriage; procedural care can be completed quickly).
- The *My Values* section includes a values clarification exercise, in which the user selects and weights (on a 0-100 scale) the relative importance of at least three of 7 decisional attributes: avoiding pain, avoiding bleeding, having the abortion at home, having an experience that feels like a miscarriage, having fewer appointments, less time off for recovery, and having a companion during the abortion.
- The *My Choice* section highlights 1 option, based on the attribute weights the user assigned in the My Values section. For instance, if a user strongly preferred to avoid bleeding and have fewer appointments, the software would suggest that a procedural abortion would be a better match. For a user who preferred having the abortion at home and having a companion present, the software would suggest that a medication abortion would be a better match. The user selects the option they prefer.
- The *Review* section asks the user to complete the 4-item SURE (Sure of Myself, Understand Information, Risk-Benefit Ratio, Encouragement) screening test [41], and advises them to talk with an expert if they answer “no” to any of the questions. This section also includes information phone lines to ensure that users can seek confidential, accurate, and nonjudgmental support.
- Lastly, in the *Next Steps* section, users see a summary of their choice and the features that matter most to them, instructions for how to save the results, keep the results private, and find an abortion health care professional. Each section of the PtDA includes a “Leave” button in case users need to navigate away from the website quickly.

We calculated readability by inputting the plain text of the web-based PtDA into a SMOG Index calculator [32], which assessed the reading level of the web-based PtDA as grade 9.2.

To ensure users’ trust in the information as accurate and unbiased we provided a data declaration on the landing page: “the clinical information presented in this decision aid comes from Society of Obstetricians and Gynaecologists best practice guidelines.” On the landing page, we also specify “This website was developed by researchers at the University of British Columbia and Dalhousie University. This tool is not supported or connected to any pharmaceutical company.”

#### Participant Characteristics

A total of 50 participants, including 25 patients and 25 health care professionals, reviewed the PtDA website and completed the survey between January and March 2021. The majority of patient (n=23, 92%) and health care professional (n=23, 92%) participants identified as cisgender women. Among patient participants, 16% (n=4) reported one or more previous abortions in various clinical settings. More than half (n=16, 64%) of health care professionals offered care in private medical offices, with other locations including sexual health clinics, community health centers, and youth clinics. Many health care professionals were family physicians (n=11, 44%), and other common types were nurse practitioners (n=7, 28%) and midwives (n=3, 12%). The mean proportion of the clinical practice of each health care professional devoted to abortion care was 18% (SD 13%). Most health care professional respondents (n=18, 72%) were involved with the provision of medication, but not procedural, abortion care. The characteristics of patient and health care professional participants are reported in Table 3.

**Table 3:** Characteristic of patient (N=25) and health care professional (N=25) survey participants.

Characteristic		Patient	Health care professionals		
**Gender, n (%)**					
	Cisgender woman	23 (92)	23 (92)		
	Cisgender man	0 (0)	1 (4)		
	Nonbinary	2 (8)	1 (4)		
**Location of abortion practice^a^, n (%)**					
	Private medical office	1 (4)	16 (64)		
	Sexual health clinic	1 (4)	4 (16)		
	Hospital	3 (12)	4 (16)		
	Other	1 (4)	6 (24)		
	Prefer not to say	4 (16)	0 (0)		
**Health care professional type, n (%)**					
	Family medicine or general practice	—^b^	11 (44)		
	Nurse practitioner	—	7 (28)		
	Midwife	—	3 (12)		
	Counsellor or social worker	—	2 (8)		
	Gynecologist	—	1 (4)		
	Other	—	1 (4)		
**Type of abortion provided, n (%)**					
	Medication-only	—	18 (72)		
	Procedural-only	—	1 (4)		
	Both	—	3 (12)		
	No response	—	3 (12)		
Percentage of work focused on abortion, mean (SD)		—	18 (13)		

^a^In total, 4 participants reported a history of abortion care, representing 6 abortion procedures.

^b^Not available.

#### Usability

The mean System Usability Score met the threshold for good usability among both patient (mean 85.7, SD 8.6) and health care professional (mean 80, SD 12) participants, although some health care professionals agreed with the statement, “I found the website to be unnecessarily complex,” (see Multimedia Appendix 3 for the full distribution of responses from patient and health care professionals). All 25 patients and 22 out of 25 (88%) health care professional respondents indicated that the user-friendliness of the PtDA was good or the best imaginable. When asked what they liked most about the PtDA, both participant groups described the ease of use, comparison of options, and the explicit values clarification exercise. When asked what they liked least about the PtDA, several health care professionals and some patients pointed out that it was difficult to use on a cell phone. A summary of usability results is presented in Table 4.

In total, 21 (84%) patients and 18 (72%) health care professionals felt that the PtDA was not missing any information needed to decide about the method of abortion in early pregnancy. While acknowledging that it is “hard to balance being easy to read/understand while including enough accurate clinical information,” several health care professionals and some patients indicated that the PtDA was too long and repetitive. Among the 4 (16%) patient participants who felt information was missing, the most common suggestion was a tool for locating an abortion health care professional. The 7 (28%) health care professionals who felt information was missing primarily made suggestions about the medical information included in the PtDA (eg, listing midwives as health care professionals with abortion care in scope of practice and the appropriateness of gender-inclusive terminology) and the accessibility of information for various language and cultural groups.

**Table 4:** Survey responses from patient (N=25) and health care professional (N=25) participants.

Survey questions (health care professional question stems indicated in brackets)		Patient	Health care professional
System Usability Scale, mean (SD)		85.7 (8.6)	80 (12)
**I would rate the overall user-friendliness of this website as, n (%):**			
	Best imaginable	8 (32)	4 (16)
	Good	17 (68)	18 (72)
	Okay	0 (0)	1 (4)
	Neutral	0 (0)	0 (0)
	Poor	0 (0)	1 (4)
	Awful	0 (0)	0 (0)
	Worst imaginable	0 (0)	0 (0)
	Missing	0 (0)	1 (4)
**Is there any information missing from the website that you feel would enhance your ability to choose (counsel your patients deciding) a method of abortion? n (%)**			
	Yes	4 (16)	7 (28)
	No	21 (84)	18 (72)
**Would this website replace or add to any existing resources that you** ***have used*** **for deciding (used with patients considering) method of abortion? n (%)**			
	Yes	10 (40)	18 (72)
	No	15 (60)	7 (28)
**When would you use the website? n (%)**			
	Before my consultation with an abortion provider	23 (92)	23 (92)
	During my consultation with an abortion provider	8 (32)	10 (40)
	After my consultation with an abortion provider	12 (48)	13 (52)
	Other	0 (0)	0 (0)
	I would prefer not to use this website	0 (0)	1 (4)
**Control preferences scale, n (%)**			
	I prefer to make the final decision about what method of abortion I will receive	6 (24)	—^a^
	I prefer to make the final selection of method of abortion after seriously considering my health care provider’s opinion	7 (28)	—
	I prefer that my health care provider share responsibility for deciding which method of abortion is best for me	4 (16)	—
	I prefer that my health care provider makes the final decision about which method of abortion will be used, but seriously considers my opinion	2 (8)	—
	I prefer to leave all decisions regarding my method of abortion to my health care provider	6 (24)	—

^a^Not available.

#### Implementation

Participants viewed the PtDA as a positive addition to current resources. Patients with a history of abortion care described looking for the information on the internet and speaking with friends, family members, and health care professionals. Compared with these sources of information, many patients liked the credibility and anonymity of the PtDA, whereas some disliked that it was less personal than a conversation. Further, 18 (72%) health care professional participants said that the PtDA would add to or replace the resources they currently use in practice. Compared with these other resources, health care professionals liked that the PtDA could be explored by patients independently and that it would support them in thinking about the option that was best for them. The disadvantages of the PtDA compared with existing resources were the length—which health care professionals felt would make it difficult to use in a clinical interaction—and the lack of localized information. In total, 23 each (92%) of patient and health care professional participants felt that they would use the PtDA before a consultation.

## Discussion

### Principal Results

We designed a web-based, interactive PtDA for the choice of method of abortion in early pregnancy [42], taking a user-centered approach that involved usability testing with 36 patients and 25 health care professionals. Both patient and health care professional participants indicated that the PtDA had good usability and would be a valuable resource for decision-making. This PtDA fills a critical need to support the autonomy of patients and shared decision-making with their health care professional related to the preference-sensitive choice of method of abortion.

### Comparison With Prior Work

A 2017 systematic review and environmental scan found that existing PtDAs for the method of abortion are of suboptimal quality [9]. Of the 50 PtDAs identified, all but one were created without expertise in decision aid design (eg, abortion services, reproductive health organizations, and consumer health information organizations); however, the development process for this UK-based pamphlet-style PtDA was not reported. The remaining PtDAs were noninteractive websites, smartphone apps, and PDFs that were not tested with users. The authors found that the information about methods of abortion was presented in a disorganized, inconsistent, and unequal way. Subsequent work has found that existing PtDAs emphasize medical (versus social, emotional, and practical) attributes, do not include values clarification, and can be biased to persuade users of a certain method [13].

To address some of the challenges identified in the literature, we systematically structured and designed elements of the PtDA following newly proposed IPDAS criteria (eg, showing positive and negative features with equal detail) [33]. We included an explicit values-clarification exercise, which a recent meta-analysis found to decrease decisional conflict and values-incongruent choices [43].

We based the decision aid on comprehensive and up-to-date scientific evidence related to the effectiveness and safety of medication abortion and procedural abortion; however, less evidence was available for nonmedical attributes. For example, many existing PtDAs incorrectly frame privacy as a “factual advantage” of medication abortion [13]. To address this, we included privacy in the About Me section as something that means “different things to different people.” Similarly, evidence suggests that patients who do not feel appropriately informed about the pain associated with their method of abortion are less satisfied with their choice [44,45]; and the degree of pain experienced varies across options and among individuals. Following the suggestion of patient participants to include stories and recognizing that evidence for the inclusion of narratives in PtDAs is emerging [46], we elected to develop brief first-person qualitative descriptions of the pain experience. The inclusion of narratives in PtDAs may be effective in supporting patients to avoid surprise and regret, to minimize affective forecasting errors, and to “visualize” their health condition or treatment experience [46]. Guided by the narrative immersion model, our goal was to provide a “real-world preview” of the pain experience [47].

In addition to integrating user perspectives on the optimal tone, content, and format of the PtDA, user testing provided evidence to inform the future implementation of the PtDA. A clear barrier to the completion of the PtDA during the clinical encounter from the health care professional perspective was its length, supporting the finding of a recent rapid realist review, which theorized that health care professionals are less likely to use long or otherwise complex PtDAs that are difficult to integrate into routine practice [48]. However, 46 out of 50 (92%) participants endorsed the use of the PtDA by the patient alone before the initial consultation, which was aligned with the patient participant’s preference to take an active role in making the final decision about their method of abortion as well as the best practice of early, pre-encounter distribution of PtDAs [48].

A unique feature of this PtDA was that it resulted from a broader program of integrated knowledge translation designed to support access to medication abortion once mifepristone became available in Canada in 2017. Guided by the principle that including knowledge users in research yields results that are more relevant and useful [49], we developed the PtDA in response to a knowledge user need, involved health care professional users as partners in our research process, including as coauthors, and integrated feedback from the expert panel. This parallels a theory of PtDA implementation that proposes that early involvement of health care professionals in PtDA development “creates a sense of ownership, increases buy-in, helps to legitimize content, and ensures the PtDA (content and delivery) is consistent with current practice” thereby increasing the likelihood of PtDA integration into routine clinical settings [48].

Viewed through an integrated knowledge translation lens, our findings point toward future areas of work to support access to abortion in Canada. Several patient participants indicated a need for tools to identify health care professionals who offer abortion care. Some shared that their primary health care professionals did not offer medication abortion despite it being within their scope of practice, and instead referred them to an abortion clinic for methods of counseling and care. We addressed this challenge in the PtDA by including links to available resources, such as confidential phone lines that link patients to health care professionals in their region. On the website we also indicated that patient users could ask their primary care providers whether they provide abortion care; however, we acknowledge that this may place the patient in a vulnerable position if their health care professional is uncomfortable with, or unable to, provide this service for any reason. Future work should investigate opportunities to shorten the pathway to this time-sensitive care, including how to support patients who use the decision aid to act on their informed preference for the method of abortion. This work may involve developing a tool for patients to talk to their primary care provider about prescribing medication abortion.

### Strengths and Limitations

Several factors affect the interpretation of our work. Although potential patient users participated in the iterative development process, the patient perspective was not represented in a formal advisory panel in the same way that the health care professional experts were. Participant characteristics collected for the think-aloud interviews demonstrated that our patient sample did not include people with lower education attainment, for whom the grade level and length of the PtDA could present a barrier [50]. Any transfer of the PtDA to jurisdictions outside Canada must consider how legal, regulatory, and other contextual factors affect the choice of the method of abortion. Since this study was completed, we have explored additional strategies to address these concerns, including additional user testing with people from equity-deserving groups, drop-down menus to adjust the level of detail, further plain language editing, and videos illustrating core content. Since the focus of this study was usability, we did not assess PtDA effectiveness, including impact on knowledge, decisional conflict, choice predisposition and decision, or concordance; however, a randomized controlled trial currently underway will measure the impact of the PtDA on these outcomes in a clinical setting. Finally, our integrated knowledge translation approach added to the robustness of our study by ensuring that health care professionals and patients were equal partners in the research process. One impact of this partnered approach is that our team has received funding support from Health Canada to implement the website on a national scale for people across Canada considering their abortion options [51].

### Conclusions

The PtDA provides people choosing a method of early abortion and their health care professionals with a resource to understand methods of abortion available in the Canadian context and support to make a values-aligned choice. We designed the PtDA using a systematic approach that included both patient and health care professional participants to help ensure its relevance and usability. Our future work will seek to evaluate the implementation of the PtDA in clinical settings, create alternate formats to enhance accessibility, and develop a sustainable update policy. We will also continue to advance access to abortion care in Canada with our broader integrated knowledge translation program of research.

## Appendix

Multimedia Appendix 1 Patient decision aid prototype.

Multimedia Appendix 2 Raw data for pain narratives.

Multimedia Appendix 3 Full distribution of System Usability Scale scores for patients and providers.

## Abbreviations

IPDAS: International Patient Decision Aid Standards
PtDA: patient decision aid
SMOG: Simple Measure of Gobbledygook
SOGC: Society of Obstetricians and Gynaecologists of Canada
SURE: Sure of Myself, Understand Information, Risk-Benefit Ratio, Encouragement

## Data Availability

Our ethics approval has specified the primary data is not available.
