# Supplementary material for: User-Centered Development of a Patient Decision Aid for Choice of Early Abortion Method: Multi-Cycle Mixed Methods Study
Source: J Med Internet Res. 2024 Apr 16;26:e48793. doi: 10.2196/48793 (PMC11061794; doi:10.2196/48793)
Supplement: Multimedia Appendix 1 [file jmir_v26i1e48793_app1.pdf]

SHOULD I CHOOSE A MEDICAL OR SURGICAL ABORTION?

	Abortion Pill<br><br>*(also called medication abortion, Mifepristone + Misoprostol, or Mifegymiso)**	Abortion Procedure<br><br>*(also called surgical abortion, or vacuum aspiration)*
**How far along in pregnancy can I be?**	Up to 10 weeks from the first day of your last period, depending on the healthcare provider.	Up to 24 or 30 weeks from the first day of your last period depending on the healthcare provider.<br><br>**The information included in this tool is about surgical abortion up to 10 weeks.**
**Who can provide the abortion?**	Family doctors, gynecologists, and nurse practitioners can prescribe medical abortion.	Gynecologists and trained family doctors can perform surgical abortions.
**Where does it happen?**	At home or in any location that feels safe and comfortable.	At a specialized clinic or hospital.
**How does it work?**	You will swallow a pill to stop the growth of the pregnancy.<br><br>About 1-2 days later, you will place other pills between your gums and cheek under your tongue, or inside your vagina to start bleeding that removes the pregnancy tissue.<br><br>[link to resource about procedure]	You will take antibiotics and be offered medications for pain and to relax.<br><br>The doctor will put freezing in your cervix and then open the it using an instrument called a dilator and possibly medications. The doctor will place a tube into the uterus and use gentle suction to remove the pregnancy tissue.<br><br>[link to resource about procedure]
**How long does it take?**	3 days on average.<br><br>For 90 in 100 people it is over after 8 days and most complete by 2 weeks.	The procedure is completed within 5-15 minutes.<br><br>You will rest in the recovery room for 30-60 minutes.

**How much will I bleed?**	Bleeding starts a few hours after you take the second pills.<br><br>Heavy bleeding usually last 2-4 hours. Depending on the length of your pregnancy, you may see blood clots or a white pregnancy sac that can get as big as an olive and may have recognizable shape.<br><br>For the first few days, the bleeding is much heavier than a regular period. Light bleeding usually continues for 2 weeks, but may last longer.	There is light bleeding similar to or less than a period.<br><br>Bleeding usually lasts for up to 2 weeks, but may last longer.
**How painful is it?**	Pain varies. During the first days it can range strong cramps that may be worse than a normal period to pain that may be like labour.<br><br>You may have some period-like cramps for a few days afterwards.<br><br>The prescriber will offer you pain medication.<br><br>[link to patient pain vignettes]	Moderate cramping happens during time of the procedure, and usually improves within 20 minutes.<br><br>You may have some period-like cramps for a few days afterwards.<br><br>The doctor will offer your pain medication for the procedure and after. In some hospitals general anesthetic is used.<br><br>[link to patient pain vignettes]
**How effective is it?**	The medication works for 95 in 100 people.<br><br>5 in 100 people may require a surgical abortion to complete the abortion.	The procedure works for 99 in 100 people.<br><br>A second procedure is rarely required.
**Is it safe?**	Very safe. Less than 1 in 100 people experience problems like getting an infection or heavy bleeding that requires treatment. transfusion because of heavy bleeding.<br><br>The risk of death is extremely small (less than 1 in 100,000).	Very safe. Less than 2 in 100 people experience problems like getting an infection, heavy bleeding that requires treatment, or a hole in the wall of the uterus (“perforation”).<br><br>The risk of death is extremely small (less than 1 in 100,000).

**What are the side effects?**	Side effects from the pain or medication include diarrhea, fever/chills, nausea, vomiting, dizziness, and headache.<br><br>These side effects usually occur over a few hours when the pregnancy is expelling from the uterus. They can be treated with other medications.	It depends on the medications given. Side effects from the medication include drowsiness, nausea, mild abdominal pain, diarrhea, and headache.
**How many appointments will I have to attend and do I have to follow-up after?**	Usually 1-3 appointments to confirm the pregnancy, get the prescription, and make sure the procedure worked.	Usually 1-2 visits to do the procedure and make sure it worked.
**How much does it cost?**	No cost if you have provincial/territorial health coverage. Details depend on province.	No cost if you have provincial/territorial health coverage. Details depend on province.
**Are there any long-term consequences on my fertility or physical health?**	There is no effect on fertility, your ability to get pregnant in the future.	There is no effect on fertility, your ability to get pregnant in the future.
**What effects can this have on my emotions/mental health?**	Emotional responses are different for each person. Abortion is not associated with an increased risk of mental illness.<br><br>[link to support resources]	Emotional responses are different for each person. Abortion is not associated with an increased risk of mental illness.<br><br>[link to patient pain vignettes]
**Will I be satisfied with my decision?**	Most people who chose medical abortion would choose to do it again.	Most people who chose surgical abortion would choose to do it again.
**How much time will it take to recover? Will I need time off from my commitments?**	1-2 days during heavy bleeding, plus time for other appointments.	The day of the procedure, plus time for other appointments.

**How hard is it to keep private from others?**	It is relatively easy to keep the abortion medications private. The bleeding can be explained as a "miscarriage."	It can be kept private, as it often requires only one visit for the procedure and minimal bleeding.<br><br>If the medications you are given cause drowsiness, you will need a ride home.
**Do I have to wait to go on contraception (birth control) after?**	You can start all hormonal contraceptives, including the vaginal ring, the day following finishing the medication.<br><br>If you want an IUD, it can be inserted as soon as the abortion is complete usually in about 1-2 weeks.<br><br>If you want an implant, it can be inserted the day you take mifepristone.	You can start all hormonal contraception, including the vaginal ring, immediately after the abortion procedure.<br><br>If you want an IUD, it can be inserted at the end of the abortion appointment.<br><br>If you want an implant, it can be inserted immediately after the abortion procedure.
**What are the key points I should remember?**	• Effective and safe for early pregnancies.<br>• Avoids sedating medications and surgical instruments being inserted into your uterus.<br>• Can happen at home, or anywhere you feel comfortable.<br>• Can take several days to complete.<br>• May feel more natural, like a miscarriage.<br>• Requires follow-up appointments.<br>• You can choose to have someone with you or to do it alone.	• Effective and safe for early pregnancies.<br>• Involves sedating medications and surgical instruments being inserted into your uterus.<br>• Happens in an appointment at a clinic or hospital.<br>• Abortion is complete once the procedure is done.<br>• May feel more like a medical procedure.<br>• Often requires few appointments and follow-up.<br>• Requires someone to drive you from the procedure.

*Sometimes, medical abortions are done with a medication called *methotrexate* instead of *mifepristone*. Using methotrexate will feel similar to using mifepristone, expect that methotrexate can be either a pill, a liquid, or an injection. Methotrexate takes longer to work than mifepristone and may not be as effective.

CAPS CPCA
Canadian Abortion Providers Support
Communauté de pratique canadienne sur l'avortement

CART-GRAC
Contraception and Abortion Research Team
Groupe de recherche sur l'avortement et la contraception

UBC

Contraception and Abortion Research Team (CART-GRAC). Early options patient decision aid. Publication date: December 20, 2019 (Version 3)
