# Supplementary material for: User-Centered Development of a Patient Decision Aid for Choice of Early Abortion Method: Multi-Cycle Mixed Methods Study
Source: J Med Internet Res. 2024 Apr 16;26:e48793. doi: 10.2196/48793 (PMC11061794; doi:10.2196/48793)
Supplement: Multimedia Appendix 2 [file jmir_v26i1e48793_app2.docx]

**Multimedia Appendix 2.** Raw data for pain experience narratives.

| **Quote** | **Type of Procedure** | **Source** | **Pain Severity Rating (1=low, 2=med, 3=high)** |
| --- | --- | --- | --- |
| "I had a medication abortion and it was basically like your heaviest period on the first day. I was nauseous for the first hour and that was it. The thing that helped me through it was when my doctor told me that if I could handle my period every month, I could handle this. It was so true- I would've never slept had he not told me that." | medical | <https://goaskalice.columbia.edu/answered-questions/does-having-abortion-hurt> | 1 |
| "I guess I felt okay physically after, it didn’t hurt for me and wasn’t as traumatic as I was expecting" | medical | <https://metro.co.uk/2019/01/09/six-women-open-up-about-what-it-was-like-to-have-an-abortion-8309559/> | 1 |
| "Other than some initial cramping, my menstruation is rarely uncomfortable. This was the same. Mild, painless cramps, no vomiting or nausea except for the lingering morning sickness. And six hours later, my nausea disappeared." | medical | <https://www.smh.com.au/lifestyle/health-and-wellness/what-its-like-to-take-the-abortion-pill-20140827-3eeyu.html> | 1 |
| "30 min prior to taking Misoprostol, I ate a rice cake to get something in my stomach, took 800mg if ibuprofen (4 OTC tabs), 500mg of acetaminophen (1 extra strength Tylenol), one 5mg/325mg hydrocodone/acetaminophen (aka Vicodin), and 4mg of ondansetron (prescription anti-nausea). I started bleeding a little an hour after finishing misoprostol, then had some clotting after 2 hrs. I found that pacing around the house (I got like 7000 steps that evening) was more comfortable than sitting or lying. When I got tired of walking around, I sat on the edge of my couch bent over a heating pad. The only other side effects I had were chills (about two hours in I was shaking and my teeth chattered for a few seconds) my boyfriend started a fire in the fireplace and it stopped. Also, oddly enough, the Misoprostol tabs (I took them buccally) left me with a sore throat and a little bit of a hoarse voice. The worst my pain/cramping ever got was like a 2/10." | medical | <https://shoutyourabortion.com/writing/positive-medical-abortion-story/> | 1 |
| "I'm relieved that this procedure, assuming it worked, was so relatively uncomplicated and painless." | medical | <https://www.guttmacher.org/journals/psrh/2003/01/having-abortion-using-mifepristone-and-home-misoprostol-qualitative-analysis> | 1 |
| "I took the mifepristone at 5 PM. Thirty-nine hours later, I took the misoprostol and got into bed with (nurse’s orders) Netflix and an arsenal of painkillers. After three hours, the only thing I felt was guilt about lying in bed instead of being productive, so I went grocery shopping. I started bleeding while I was wheeling past the canned goods and I thought about how I’d heard that Mifegymiso is helpful to women in abusive relationships because it mimics a miscarriage.  The bleeding continued, like a heavy period, for four days. I felt bloated, but was otherwise surprised to find I felt good. I worked. I talked to friends. I went to the gym. I felt strong. I felt like I was in control of my life. " "On the fifth day, more intense cramps passed a clump of tissue half the size of a dried apricot. Up to that point, it had mostly been bleeding. I texted the nurse." | medical | <https://www.vice.com/en_ca/article/zmaqwy/my-abortion-was-the-most-positive-experience-i-had-with-canadian-health-care> | 1 |
| "There was a gradual increasing of cramps and bleeding, but none of if felt any worse than a bad period. I never took anything for the pain or discomfort." | medical | <https://www.cosmopolitan.com/sex-love/a3389296/abortion-pill-mifeprex-mifepristone/> | 2 |
| "I took the 4 pills you dissolve in your cheeks exactly 24 hours later at home and laid in bed with my dog & boyfriend. I was doing good until I puked 45 minutes later. I panicked. I immediately called PP and asked them what to do. They said it was okay as long as I started bleeding soon and to not worry because most of it had already dissolved. I did start bleeding and relief fell over me. It was heavy the first couple of hours and the cramps were a 6/10 on the pain scale. I had a few clots in the beginning but nothing like I expected." | medical | <https://shoutyourabortion.com/writing/i-had-a-medical-abortion-and-a-surgical-abortion-you-can-do-this/> | 2 |
| "I took my pain pills and waited then I took my 4 pills, waited the thirty minutes then washed them down with water.  About an hour later I started get cramps and things started going. I didn’t really bleed into my pad in the beginning, everything seemed to happen when I was on the toilet. I ended up having bad diarrhea too. I think I knew when I passed it, it was a clot about the size of a lemon. I was able to actually get some rest and the cramps only came in waves really. It’s now the day after I took all the pills (I called into work) and I have no more nausea, the bleeding is like a normal period. Mostly I am tired....I was so scared that the pain would be horrible. But honestly I have had period much worse than this. Much worse. I had some chills, some severe nausea (I use breathing tricks to not vomit) and cramps that only came in waves. If anyone who is as worried as I was; you got this!" | medical | <https://shoutyourabortion.com/writing/my-8-week-abortion/> | 2 |
| "The second time was early enough to bring the pills home. There was a lot of cramping. I was nauseated after." | medical | <https://www.romper.com/p/9-women-describe-what-it-really-feels-like-to-have-abortion-32253> | 2 |
| "Thankfully my regular doctor was able to prescribe me the abortion pill. It was just like a heavy period." | medical | <https://www.buzzfeed.com/caseygueren/heres-what-44-women-want-you-to-know-about-their-abortions> | 2 |
| "The procedure itself was uncomfortable but not incredibly painful." | medical | <https://www.buzzfeed.com/caseygueren/heres-what-44-women-want-you-to-know-about-their-abortions> | 2 |
| "Now that the experience is over, I feel relieved. The scariest part of the whole thing was not really knowing what to expect from my body. Everything went smoothly. I did experience a lot of pain for about five hours, but that was it." | medical | <https://www.guttmacher.org/journals/psrh/2003/01/having-abortion-using-mifepristone-and-home-misoprostol-qualitative-analysis> | 2 |
| "I mean, it was painful, but that's to be expected....I don't think it was really a terrible, terrible experience. You know what I mean. I didn't die or [have] anything major happen....I was expecting like my worst period, and this was just like phenomenal....for three hours...I took two more painkillers, and I went back to bed." | medical | <https://www.guttmacher.org/journals/psrh/2003/01/having-abortion-using-mifepristone-and-home-misoprostol-qualitative-analysis> | 2 |
| " The night I took them, I covered all the bases of physical misery: I bled. I was nauseous. I had cramps. I could not get comfortable. I couldn’t read. I couldn’t watch TV. I could only thrash around and fail to imagine ever feeling better. (Thank God for marijuana, which was helpful.) I decided right there that I’d prefer carrying a baby to term, raising him up from infancy all the way to the proud moment where I’d give him a nail-studded club with which to stave off the fascists fighting to take control of our family water supply, than go another round with RU-486, which is basically ayahuasca minus the spirit animals. " | medical | <https://www.thecut.com/2019/06/the-best-abortion-ever.html> | 2 |
| "The first pill I had very, very light cramping on Friday night and Saturday, but no bleeding. Sunday evening I started to spot and also passed a bit of a brown slimy substance when I went to the toilet.  I got home 30 mins later and took two co-codamol tablets and laid in bed. For the first two hours, I felt nothing and then light cramping started to step in. About an hour later this light cramping turned into heavy period pain cramping, but this started to get worse and worse... I went to the toilet where I had light diarrhoea, but not a lot of bleeding. I sat here for a while as it felt more comfortable.  I kept having hot and cold flushes as the pain intensified - but it hadn't been long enough to take more pain relief. The pain was excruciating with no respite. The pain then got so bad that I could only scream in agony. There was no position that I could get in to give me any relief. The pain was like nothing I've ever felt before. It was excruciating and with no respite - like severe period pains, but 100,000 x worse and sharper, and also a lot lower down. I couldn't bear it anymore and took two more co-codamol tablets, even though four hours hadn't yet elapsed, but yet I was still in excruciating agony."  "As I wasn't bleeding that much (like a light to normal period) I was getting worried that I would have to endure this for many more hours - and I just couldn't. When I stood up I almost passed out with the pain" | medical | <https://www.2plusabortions.com/our-stories/2020/1/25/i-want-to-share-my-experience-of-having-a-medical-abortion-compared-to-a-surgical-abortion-i-had-5-years-ago> | 3 |
| "I had a medical abortion, so once I’d been given the pills, I was sent home. It was more painful than I expected (the worst pain I’ve ever experienced), and I imagine it would be awful to go through that alone." | medical | <https://www.romper.com/p/9-women-describe-what-it-really-feels-like-to-have-abortion-32253> | 3 |
| "The procedure itself was very simple. Just the pills to take. Afterwards I felt like I should have had pain, but up to 24 hours later there was no pain. Then I started feeling guilty about it because I felt as though this was a big deal and I deserved to face some pain because I did it. Well, the pain came two days later and I thought I was dying. It was not normal at all. Turns out that the abortion medication, which basically is supposed to cause you to shed the lining, etc. also exacerbated my fibroid pain. I didn't know I had fibroids before." | medical | <https://www.romper.com/p/9-women-describe-what-it-really-feels-like-to-have-abortion-32253> | 3 |
| "So the next day I crawled into bed with my boyfriend, put on my super cool adult diapers, inserted the pills, and put on some Game of Thrones. And let me fucking tell you, it was excruciating, like... I honestly believed I could go into shock kind of pain. These cramps radiated throughout my whole body to the point where I could barely breathe. I actually cut my palms on my nails from clenching my fists so hard. But once it was done, it was done." | medical | <https://www.buzzfeed.com/caseygueren/heres-what-44-women-want-you-to-know-about-their-abortions> | 3 |
| "I had a medical abortion. I remember the ultrasound and the nurse asking me if I wanted to look at the screen. It was weird seeing the little dot on the screen. I took the first pills, drove home, and waited until the next day for the next dose. I felt like I was dying. Imagine the worst period of your life and multiply it by 100. I lay on the cold floor of my bathroom for three hours because I couldn't get comfortable anywhere else." | medical | <https://www.buzzfeed.com/caseygueren/heres-what-44-women-want-you-to-know-about-their-abortions> | 3 |
| "I was driven to my appointment a week later, had an ultrasound, and I received my first dose of the medication; I cried the entire way home. That night after my second dose, I was overcome by my the pain and vomited over and over again. I begged my then-boyfriend to take me to the ER because the pain was so terrible. After several hours, the pain subsided and I finally fell asleep." | medical | <https://www.buzzfeed.com/caseygueren/heres-what-44-women-want-you-to-know-about-their-abortions> | 3 |
| "Within an hour, I experienced the worst cramps I have ever felt. When the crippling pain subsided enough, I got up to go to the bathroom and put a pad on. As I was putting the pad on, I looked down and saw what I can only describe as the single most horrific sight. I watched my fetus still in its sac fall out of me into the toilet. All I could do was scream and sob." | medical | <https://www.buzzfeed.com/caseygueren/heres-what-44-women-want-you-to-know-about-their-abortions> | 3 |
| "Truthfully the actual abortion was incredibly painful. But the entire experience was much simpler, quicker and easier than you’d expect. ‘I was taken into a small room, asked questions about if it was my choice and was I sure this was what I wanted, I said yes to everything. ‘I was then given a tablet to swallow, then I was put straight on to the contraceptive injection. The next day we had to go back, to another small room, I had to lay on a bed, the nurse inserted two tablets into me and I was then sent home with painkillers." | medical | <https://metro.co.uk/2019/01/09/six-women-open-up-about-what-it-was-like-to-have-an-abortion-8309559/> | 3 |
| "This procedure was exactly what I needed at the time, so I don't really feel great saying anything negative about it. That said, I would be dishonest if I didn't say that I experienced some of the worst pain of my life during my medical abortion. All the pain came from the cramps; they have to push out all your uterine lining and the embryo, so they are very powerful. They felt like a worse version of the debilitating menstrual cramps I used to deal with in high school. I felt sweaty, a little woozy, and in agonizing pain." "Did I mention the cramping? Because you don't just cramp; you cramp. My cramps began after I took the misoprostol. After some time, they developed into the most painful menstrual-type cramps I had ever experienced." | medical | <https://www.bustle.com/articles/122877-what-getting-a-medical-abortion-was-like-for-me> | 3 |
| "The next day I dutifully took the second round of pills and left them between my cheek and my teeth, as instructed. They dissolved and I didn't feel anything. Waiting for whatever was supposed to happen next was a terrifying brand of anxiety. About a half-hour later, I started to cramp up—first, a sort of palatable, PMS-like ache and then the worst pain I've ever felt in my life. I got dizzy and slid off my brother's bed onto the floor for fear I might faint. This went on for a few hours, with the cramping subsiding slightly as the pain medication I was prescribed kicked in. I was bleeding a lot. I'd fill up a jumbo-size pad and moved on to the next every hour until I fell asleep that night. I couldn't eat. I could barely move." | medical | <https://www.byrdie.com/abortion-stories> | 3 |
| Minimal pain:  "I never had an abortion, so I was expecting more pain but there was no pain. It was just normal; I was doing the house chores I am used to doing” "It wasn’t painful at all, not even a little. There was no such feeling of pain. Only when the pregnancy started to discharge, there was a feeling of something coming out, like when I’m on my period … but there was absolutely no pain”  Brief intense pain: “It hurt terribly, it hurt in a way that it’s very unlike any normal pain … I had 30 minutes of intense pain, after that I felt better, then gradually it eased and then I could walk as usual”   Intermittent pain: “It pained and disappeared and again pained and again disappeared” “I think it’s like labor pain, intermittent pain from light pain to heavy pain”   Constant pain: “The pains were like 10 … for about 3 or 4 hours … there was no change. The pains were constant”   Other: “I felt really cold, and I was shivering even when I was staying in the sun, it still felt cold”  “Diarrhea was the most intolerable. It made my stomach gurgle and I felt nauseous so I needed to go to the bathroom constantly even though I felt cold and just wanted to stay in my bed … the diarrhea and nausea were the worst”  “I tolerated the pain for half an hour, it was all because of dizziness that I couldn’t tolerate the pain”   “…it felt so numb with pain…I think it was because of the pills melting. I think they made me feel that way”  "…There was no abdominal pain. Only my limbs, it felt like I can’t handle it anymore, I felt paralyzed…It hurt terribly, it hurt in a way that it’s very unlike any normal pain, I have never experienced such kind of pain. My limbs couldn’t even move” | medical | <https://bmcwomenshealth.biomedcentral.com/articles/10.1186/s12905-019-0816-0> |  |
| "The first pill I was given at the clinic on my first visit. I didn’t experience any side effects or any symptoms after the first pill. The second set of 4 pills I was instructed to take at home 24-48 hours after the first pill. After 36hrs, I took ibuprofen & anti-nausea medication and 1 hour after that I took the second set of four pills." "Within 1 hour after taking the four pills, I experienced painful low abdominal/pelvic area cramping & within 4 hours I was soaked in blood." "At 4 weeks post conception, medication abortion felt mostly like a regular menstrual period except for heavier & longer days bleeding and the initial painful cramping. I experienced bleeding for 7 days and very tiny amount for additional 3 days." "For 5 weeks after abortion, I continued to have mild period-like low abdomen/pelvic area cramping on and off. I also continued to have breast tenderness for 5 weeks after the abortion." | medical | <https://shoutyourabortion.com/writing/medication-abortion/> |  |
| "The procedure was quick and almost painless. I felt slight discomfort but it really wasn't anything worse than an extended pap smear" | surgical | <https://www.cosmopolitan.com/sex-love/news/a31727/what-its-really-like-to-have-an-abortion/> | 1 |
| "The worse bit was removing the pregnancy. They use a vacuum device which is quite noisy. It wasn't painful, but I could feel what was going on." | surgical | <https://www.2plusabortions.com/our-stories/2020/1/25/i-want-to-share-my-experience-of-having-a-medical-abortion-compared-to-a-surgical-abortion-i-had-5-years-ago> | 1 |
| "My abortion happened at the juncture between the first and second trimesters, and although it was a short outpatient procedure, my Canadian-trained doctor opted for conscious sedation like that used during a colonoscopy. So, no pain. I remember, as I was regaining my senses, seeing a small jar with blood in it, the remains of my gestational sac and placenta, but I don’t remember physical discomfort. I must have been sore in the days that followed, but everything else I was processing far outweighed any physical discomfort." | surgical | <https://www.bustle.com/p/abortion-ama-does-abortion-hurt-7923840> | 1 |
| "I had a D&C to terminate a pregnancy at 10 weeks. Before the procedure, I was given laughing gas, so the only sensation I experienced was a slight tugging in my uterus. Afterwards, I had cramping that lasted for about two days, but it wasn’t more painful than what I feel during a heavy period. After my abortion I went on to have a healthy pregnancy and gave birth to a beautiful baby girl, now three years old." | surgical | <https://www.bustle.com/p/abortion-ama-does-abortion-hurt-7923840> | 1 |
| "The nurse then told me that they was going to place a mask over my face and insert the anesthetic through the cannula and how it might feel. She held my hand and they placed the mask over my face, they told me to take deep breaths and I did. That’s honestly the last thing i remember. I don’t remember any pain, any discomfort or any of the procedure." | surgical | <https://shoutyourabortion.com/writing/my-surgical-abortion-at-12-weeks/> | 1 |
| "The procedure was quick, relatively painless, and felt like a somewhat-extended pap smear. " | surgical | <https://www.romper.com/p/9-women-describe-what-it-really-feels-like-to-have-abortion-32253> | 1 |
| "The doctor did a D&C that day. I got an infection afterward, so the experience wasn’t a walk in the park, but I don’t remember the actual procedure being terrible. " | surgical | <https://www.health.com/condition/pregnancy/women-share-abortion-stories> | 1 |
| "There’s nothing to compare it to. It hurt slightly, but they'd numbed me. The feeling was of some pressure, and recovery was brief and not bad." | surgical | <https://www.health.com/condition/pregnancy/women-share-abortion-stories> | 1 |
| "The procedure was uneventful, and once again recovery was easy." | surgical | <https://www.teenvogue.com/story/abortion-stories> | 1 |
| "I opted for the procedure, the wait was several hours long but the procedure turned out to not be as painful as I had thought." | surgical | <https://www.teenvogue.com/story/abortion-stories> | 1 |
| "I had to have a speculum inserted (if you've ever had a smear or coil fitted you'll know what this is like), but as they need to open up your cervix I found it extremely uncomfortable. They then applied a numbing gel and injected a localised anesthetic - this was less painful than I was anticipating, only a sharp jab." | surgical | <https://www.2plusabortions.com/our-stories/2020/1/25/i-want-to-share-my-experience-of-having-a-medical-abortion-compared-to-a-surgical-abortion-i-had-5-years-ago> | 2 |
| "My pregnancy was around nine weeks, and I opted for a standard surgical abortion. I had conscious sedation during the procedure, so I was a little out of it, but I do remember feeling sharp pains for a few minutes. By the time I was dressed and in the recovery room, I was only having dull cramps that felt like moderate period cramps. Surprisingly, I didn't have much cramping at all once I got home. The only discomfort I had was in my cervix and vaginal wall; it felt like I had a paper cut for a few days. I got a rash from the adhesive on the bandaid the clinic put on my IV site and that was the biggest issue with my recovery.  I had an IUD (intrauterine device that prevents pregnancy) inserted a month after my abortion, and I remember being surprised that the IUD was much more painful." | surgical | <https://www.bustle.com/p/abortion-ama-does-abortion-hurt-7923840> | 2 |
| "The physical experience of my abortion was that I was given medications that would allow me to feel relaxed and intravenous medication to aid any pain or discomfort management. The medications ... were great because my pelvic floor and glutes were relaxed throughout the procedure. Regardless of the medication used or the lidocaine used to soften the cervix for dilation, I still felt manageable cramps during both the dilation and procedure.  After my abortion, I asked to have a hormonal IUD inserted. I recall the cramping from the IUD more clearly than the discomfort of the dilation and abortion: it felt like a short but consecutive wave of cramps. My procedure lasted less than three minutes — softening of the cervix, dilation, aspiration — although, at the time, I felt time moving very slowly. My adrenaline was still high, my body was hyper-alert, [and] my focus was on the possible experience of pain, which never came in the form of pain but in the form of cramps and discomfort.  I would say my strongest cramp came from the insertion of the IUD, more so than from the abortion itself." | surgical | <https://www.bustle.com/p/abortion-ama-does-abortion-hurt-7923840> | 2 |
| "I had a surgical aspiration at seven weeks with conscious sedation. During the procedure, it mostly felt like intense pressure; like my dachshund was sitting right on top of my pelvis. It was rather quick. Most of the pain I experienced was after the procedure, but the pain I experienced while I was recovering felt very much similar to a really bad period, but not the worst I've ever had. (I have polycystic ovary syndrome, also known as PCOS.) I know many people have different experiences with their abortions in relations to pain. For me, my pain tolerance is pretty high, and I'm used to the pain I experienced." | surgical | <https://www.bustle.com/p/abortion-ama-does-abortion-hurt-7923840> | 2 |
| "My own abortion experience wasn't particularly painful. It was as if I was experiencing five days of cramps all at once and for only a few moments. There was some lingering discomfort, but it didn't last beyond a few days. I had a surgical abortion, so my cervix was numbed, then dilated, then a plastic tube was inserted into my uterus to suck out the contents. I was administered a light sedative prior to the procedure to minimize cramping and so I could relax while remaining conscious. After the abortion was over — it took about seven minutes from start to finish — I was given a heating pad, antibiotics to help prevent infection, and some crackers and juice. I was in the recovery room for about 20 minutes before I felt ready to get up, walk out of the clinic, and go home.  I was never in an immense amount of pain, and I was never more sure of my decision. And even at my most uncomfortable, I was comforted by the fact that I knew I was making the best choice for myself, my body, and my future." | surgical | <https://www.bustle.com/p/abortion-ama-does-abortion-hurt-7923840> | 2 |
| "The doc inserted something into me, then said “here comes the shots”. Those seemed painful at the time, but in retrospect my nerves made it worse. I’d rate them a 5 out of 10. He then dilated me and I heard one or two clicks from the device. That was more uncomfortable than painful. I’d rate them a 3 or 4 on the pain scale. And, that pain only lasted while the clicking happened, maybe 10 or 15 seconds. Then he used what looked like a turkey baster (the nurse showed me it before, I couldn’t see) and I heard a weird ‘sloop’. Maybe a tiny tug sensation came with it, but barely noticeable and I may have imagined it because I read it in other woman’s stories. " "Its been 2 days since then, and the “sore uterus” feeling is pretty much all I’ve dealt with since. Maybe some very very light cramping a few seconds a day, but hardly noticeable. I’ve been taking it easy and have had maybe 2 tablespoons of bleeding since the procedure" | surgical | <https://shoutyourabortion.com/writing/youre-scared-will-hurt-like-read/> | 2 |
| "I go into the recovery area I take a one time antibiotic before the procedure along with just two high dose ibuprofen and wait 20 minutes, then during the suction abortion they put my feet and legs in stirrups, one lady is doing the procedure with the speculum and one other doctor is holding my hand telling me to breathe and telling me what to expect next, and honestly even though the pain was intense, the whole thing with me being 6 weeks along only took maybe 5 minutes, it was so fast, the pain made me immensely sweaty, dizzy and ears felt plugged but that only lasted two minutes at most while I was in the recovery room, then that wore off and then I just felt strong period cramps and I gotta be honest it was seriously a lot better than I expected, seriously I was so relieved and I didn’t have bad side effects" "I feel NORMAL again, have felt normal since after the procedure, I recovered quickly without sedation or hard narcotics, so far I’ve only had slight cramps and some brown spotting but that’s it" | surgical | <https://shoutyourabortion.com/writing/better-than-i-expected/> | 2 |
| "I got a surgical abortion. Basically, they used what resembled a giant syringe, but without the needle, to “suck” it out. It felt like a strong period cramp for 5-10 seconds, and then it was done. " | surgical | <https://shoutyourabortion.com/writing/my-5-week-5-day-abortion/> | 2 |
| "It felt like cramps. I did a very early one... It hurt like very bad period cramps, which was normal for me." | surgical | <https://www.romper.com/p/9-women-describe-what-it-really-feels-like-to-have-abortion-32253> | 2 |
| "The procedure itself only took about two minutes. It hurt quite a lot, but it didn't last long. Maybe I had it easy, but I didn't have a whole lot of cramping. The nurse escorted me to the recovery room and got me a couple cookies and a soda. " | surgical | <https://www.buzzfeed.com/caseygueren/heres-what-44-women-want-you-to-know-about-their-abortions> | 2 |
| "I had local anesthesia, and they talked me through everything they were doing. The dilating of the cervix was probably the worst part not surprisingly. I’d taken the next day off because I had no idea if the recovery was going to bad or not—it felt like a really, really heavy period, with lots of cramping." | surgical | <https://www.health.com/condition/pregnancy/women-share-abortion-stories> | 2 |
| "I went to all my appointments alone, including the procedure itself. I had the option of IV sedation but I had no one to drive me home, so I had to go with just Ibuprofen. The procedure was not that painful to me—it was the equivalent of having the worst period cramps you can imagine. It was most painful when they dilated my cervix, but the suction itself was not bad." | surgical | <https://www.health.com/condition/pregnancy/women-share-abortion-stories> | 2 |
| "It felt like a strong period cramp. It was not pleasant. But I would definitely do it again if I had to. " | surgical | <https://www.teenvogue.com/story/abortion-stories> | 2 |
| "They laid a heat pad over my stomach before dilating my cervix, and I honestly barely even felt the numbing shots. However the next few minutes consisted of terrible cramps that seemed never ending. The lady next to me kept reminding me to breathe and told me to squeeze her hand, and she told me to hold on because I was almost done. I really hadn’t known how my body would react, but I rarely get cramps so it felt terrible for me. I also felt a lot of pressure. On a scale of 1 to 10, I’d say the cramps were a 6 or 7 (with 10 being most painful). Of course, every person reacts differently and I have a low pain threshold. Near the end, I also began feeling nauseous." "The cramps got a little more intense near the end, but the doctor told me she was just checking to make sure she got everything . . ." | surgical | <https://exhaleprovoice.org/post/surgical-abortion-9-weeks-2-days/> | 3 |
| "She said you are going to experience 10 seconds of period cramps 3 times. We counted and the cramps were pretty bad. 8/10. And we as soon as we finished the 3rd round of cramps she yelled, “sweetie you’re done! You did it!” I was relieved. " "It’s been a little under a week since my procedure. I am still bleeding/spotting, but I have had little to no cramps." | surgical | <https://shoutyourabortion.com/writing/i-had-a-medical-abortion-and-a-surgical-abortion-you-can-do-this/> | 3 |
| "Before the procedure, I was given a saline lock of Fentanyl to relax me and induce drowsiness, which affected me immediately. The surgeon then inserted a speculum, which was a new and admittedly painful experience for me, and injected my cervix with the local anesthetic. While there was a few excruciating seconds, they were short lived and manageable. Next, my cervix was dilated with sterile rods, which in terms of pain was similar to the needle before being anesthetized as it was being inserted. The actual vacuum aspiration was very similar to bad period cramps, which lasted for about 2 minutes and then the procedure was over, with the doctor confident that everything went smoothly. I got my IUD installed at that point but all I felt was a relatively minor sting" "At the worst points in the procedure, I would rank my pain about an 8 but those were only about four 2 second intervals, the majority of the procedure (which was maximum 5 minutes) was maybe a 5. I was told that I have quite a narrow and short vaginal canal, it should be even less painful for most women. " | surgical | <https://www.quora.com/What-does-it-feel-like-to-have-an-abortion> | 3 |
| "In a minute or so, she said, “Okay, now instrument.” There was a feeling between uncanny and mildly unpleasant, then there was pain. It was like the worst cramp ever times three, but not worse than that." | surgical | <https://www.thecut.com/2019/06/the-best-abortion-ever.html> | 3 |
