# Supplementary material for: User-Centered Development of a Patient Decision Aid for Choice of Early Abortion Method: Multi-Cycle Mixed Methods Study
Source: J Med Internet Res. 2024 Apr 16;26:e48793. doi: 10.2196/48793 (PMC11061794; doi:10.2196/48793)
Supplement: Multimedia Appendix 3 [file jmir_v26i1e48793_app3.docx]

**Multimedia Appendix 3.** Full distribution of System Usability Scale scores for patients and providers.

| **Survey questions**  **(provider question stems indicated in brackets)** | **Patient (N=25)** | **Provider (N=25)** |
| --- | --- | --- |
|  | Mean ± SD  N (%) | Mean ± SD  N (%) |
| I would like to use the website |  |  |
| Strongly agree | 17 (68) | 10 (40) |
| Agree | 8 (32) | 13 (52) |
| Neutral | 0 (0) | 1 (4) |
| Disagree | 0 (0) | 1 (4) |
| Strongly disagree | 0 (0) | 0 (0) |
| I find the website unnecessarily complex |  |  |
| Strongly agree | 0 (0) | 1 (4) |
| Agree | 0 (0) | 2 (8) |
| Neutral | 0 (0) | 3 (12) |
| Disagree | 17 (68) | 16 (64) |
| Strongly disagree | 8 (32) | 3 (12) |
| I think the website is easy to use |  |  |
| Strongly agree | 15 (60) | 7 (28) |
| Agree | 10 (40) | 16 (64) |
| Neutral | 0 (0) | 1 (4) |
| Disagree | 0 (0) | 1 (4) |
| Strongly disagree | 0 (0) | 0 (0) |
| I would need the support of an expert to be able to use the website |  |  |
| Strongly agree | 0 (0) | 0 (0) |
| Agree | 0 (0) | 0 (0) |
| Neutral | 0 (0) | 1 (4) |
| Disagree | 11 (44) | 9 (36) |
| Strongly disagree | 14 (68) | 15 (60) |
| I find the various components of the website well-integrated |  |  |
| Strongly agree | 9 (36) | 2 (8) |
| Agree | 16 (64) | 20 (80) |
| Neutral | 0 (0) | 1 (4) |
| Disagree | 0 (0) | 2 (8) |
| Strongly disagree | 0 (0) | 0 (0) |
| I think the website is too inconsistent |  |  |
| Strongly agree | 0 (0) | 0 (0) |
| Agree | 0 (0) | 0 (0) |
| Neutral | 0 (0) | 1 (4) |
| Disagree | 13 (52) | 14 (56) |
| Strongly disagree | 12 (48) | 8 (32) |
| I imagine most people could learn to use the website very quickly |  |  |
| Strongly agree | 9 (36) | 5 (20) |
| Agree | 15 (60) | 19 (76) |
| Neutral | 1 (4) | 0 (0) |
| Disagree | 0 (0) | 1 (4) |
| Strongly disagree | 0 (0) | 0 (0) |
| I find the website very awkward to use |  |  |
| Strongly agree | 0 (0) | 0 (0) |
| Agree | 0 (0) | 0 (0) |
| Neutral | 1 (4) | 5 (20) |
| Disagree | 15 (60) | 13 (52) |
| Strongly disagree | 9 (36) | 7 (28) |
| I feel very confident using the website |  |  |
| Strongly agree | 9 (36) | 7 (28) |
| Agree | 13 (52) | 16 (64) |
| Neutral | 2 (8) | 1 (4) |
| Disagree | 0 (0) | 1 (4) |
| Strongly disagree | 0 (0) | 0 (0) |
| Missing | 1 (4) | 0 (0) |
| I need to learn a lot of things before I start using the website |  |  |
| Strongly agree | 0 (0) | 0 (0) |
| Agree | 0 (0) | 0 (0) |
| Neutral | 0 (0) | 1 (4) |
| Disagree | 13 (52) | 9 (36) |
| Strongly disagree | 12 (48) | 14 (56) |
